# Fighting Antibiotic-Resistant Bacterial Infections by Surface Biofunctionalization of 3D-Printed Porous Titanium Implants with Reduced Graphene Oxide and Silver Nanoparticles

**DOI:** 10.3390/ijms23169204

**Published:** 2022-08-16

**Authors:** Hongshan San, Marianne Paresoglou, Michelle Minneboo, Ingmar A. J. van Hengel, Aytac Yilmaz, Yaiza Gonzalez-Garcia, Ad C. Fluit, Peter-Leon Hagedoorn, Lidy E. Fratila-Apachitei, Iulian Apachitei, Amir A. Zadpoor

**Affiliations:** 1 Department of Biomechanical Engineering, Faculty of Mechanical, Maritime and Materials Engineering, Delft University of Technology, Mekelweg 2, 2628 CD Delft, The Netherlands; 2 School of Materials Science and Engineering, Henan Polytechnic University, Jiaozuo 454000, China; 3 Department of Materials Science and Engineering, Faculty of Mechanical, Maritime and Materials Engineering, Delft University of Technology, Mekelweg 2, 2628 CD Delft, The Netherlands; 4 Department of Medical Microbiology, University Medical Center Utrecht, Heidelberglaan 100, 3584 CX Utrecht, The Netherlands; 5 Department of Biotechnology, Faculty of Applied Sciences, Delft University of Technology, Van der Maasweg 9, 2629 HZ Delft, The Netherlands

**Keywords:** antibiotic-resistant bacteria, reduced graphene oxide, silver nanoparticles, plasma electrolytic oxidation, implant-associated infections, titanium, 3D printing

## Abstract

Nanoparticles (NPs) have high multifunctional potential to simultaneously enhance implant osseointegration and prevent infections caused by antibiotic-resistant bacteria. Here, we present the first report on using plasma electrolytic oxidation (PEO) to incorporate different combinations of reduced graphene oxide (rGO) and silver (Ag) NPs on additively manufactured geometrically ordered volume-porous titanium implants. The rGO nanosheets were mainly embedded parallel with the PEO surfaces. However, the formation of ‘nano-knife’ structures (particles embedded perpendicularly to the implant surfaces) was also found around the pores of the PEO layers. Enhanced in vitro antibacterial activity against methicillin-resistant *Staphylococcus aureus* was observed for the rGO+Ag-containing surfaces compared to the PEO surfaces prepared only with AgNPs. This was caused by a significant improvement in the generation of reactive oxygen species, higher levels of Ag^+^ release, and the presence of rGO ‘nano-knife’ structures. In addition, the implants developed in this study stimulated the metabolic activity and osteogenic differentiation of MC3T3-E1 preosteoblast cells compared to the PEO surfaces without nanoparticles. Therefore, the PEO titanium surfaces incorporating controlled levels of rGO+Ag nanoparticles have high clinical potential as multifunctional surfaces for 3D-printed orthopaedic implants.

## 1. Introduction

Additive manufacturing (AM), also known as 3D printing, is revolutionizing the manufacture of orthopaedic implants. It is utilized to better satisfy patient-specific bone reconstruction requirements [1]. Stress shielding and poor osseointegration of solid titanium implants can be prevented by using the AM process to fabricate volume-porous implants, which favor tissue ingrowth [2,3,4,5]. The porous structure is also suitable to transport nutrients, to expel the metabolic wastes of cells, and to faster integrate the implant with the surrounding bone tissue [6,7,8]. However, the high surface area of AM implants may increase the risk of bacterial contamination [9]. Therefore, developing an antibacterial function to prevent bacterial adhesion and biofilm formation presents a major challenge for these implants. The antibacterial function becomes vital for future implants as the number of antibiotic-resistant bacterial infections is predicted to significantly increase in the near future [10].

Reduced graphene oxide (rGO) is a dense honeycomb structure (sheet-like shape) consisting of monolayers of carbon atoms with oxygen-containing functional groups at the edges [11]. It has unique physicochemical characteristics, including high electrical conductivity, good dispersibility and stability in aqueous solutions, resistance to degradation, and high mechanical strength [11,12]. The distinct properties of the surface and edges of rGO inside living systems make it a unique and novel biomaterial [13,14]. However, this attribute is fraught with uncertainties when applied in different conditions and forms (e.g., nanosheets or free-standing paper) and causes cytocompatibility concerns [15,16,17,18,19]. Various mechanisms have been proposed to assess the antibacterial properties of rGO materials, which can be generally classified into chemical and physical damages. Reactive oxygen species (ROS) generated by rGO have been considered the primary cause of chemical damage to bacteria. ROS are highly reactive molecules and free radicals containing oxygen, such as superoxides, hydroxyl radicals, and peroxides, which can cause oxidative stress that kills bacteria [20,21,22,23,24,25]. In addition, rGO has been reported to damage bacteria physically by acting as a nano-knife. The sharp edges of the rGO sheets may cut cell walls and kill bacteria [26,27]. Another advantage of rGO is that its high surface area has a high adsorption capacity for other compounds, which can act against bacteria and give rise to novel combinatorial properties after mixing [27,28,29,30,31]. Amongst these compounds, silver nanoparticles (AgNPs) are very attractive due to their high antibacterial activity against a broad spectrum of bacteria and low bacterial resistance [32,33,34].

In addition to their antibacterial properties, it has been shown that rGO could be used in biomaterial systems to stimulate the osteogenic differentiation of stem cells [35,36]. Enhanced osteogenic differentiation improves the osseointegration of implants and could further decrease the risk of implant-associated infections. Therefore, the application of rGO has the potential to afford AM implants with multifunctional surface properties.

In light of the above, the mixture of rGO and AgNPs may lead to enhanced antibacterial activity relative to single components while also improving the osteogenic differentiation of progenitor cells. Recent studies on rGO/AgNP nanocomposites have reported improved antibacterial properties with respect to both Gram-positive and Gram-negative bacteria [37,38]. Plasma electrolytic oxidation (PEO) is an effective, single-step surface modification process for the incorporation of nanoparticles into the surfaces of light metals [39,40]. Furthermore, it is a suitable method for preparing interconnected porous oxide layers on complex surfaces fabricated by AM [1,41,42]. There are only a few reports on PEO layers with graphene-based nanosheets, the general aim of which was to improve the mechanical properties of the oxide layer on Al and Mg alloys for engineering applications [43,44,45]. In this study, PEO layers incorporating rGO+Ag nanoparticles were produced and characterized for the first time. They were grown on the surface of volume-porous AM titanium implants made from Ti-6Al-4V. The morphological and physicochemical properties of the resulting surfaces, their antibacterial behavior, the cytocompatibility of the implants, and their osteogenic potential were evaluated using a broad range of assays.

## 2. Materials and Methods

### 2.1. Nanoparticles

The rGO stacked in 10–15 sheets, each with 1 nm average thickness and an average size between 500 and 800 nm (Sigma-Aldrich, Zwijndrecht, The Netherlands), were dispersed in isopropanol (Sigma-Aldrich, Zwijndrecht, The Netherlands) by ultrasonication for 3 h to mechanically exfoliate the as-received material to a few layers of rGO. The rGO suspension was thereafter magnetically stirred under ambient conditions until the liquid was evaporated entirely. Silver nanoparticles (Sigma-Aldrich, St. Louis, MO, USA) were added to the rGO suspension and were ultrasonicated for an additional 10 min to prepare the rGO+Ag nanoparticles. The AgNPs had a spherical shape with a diameter of 7–25 nm [46].

### 2.2. Synthesis of the Antibacterial Surfaces

The volume-porous AM Ti-6Al-4V samples used for PEO treatment were 0.5 mm in diameter and 4 cm in length. They were rationally designed and produced using the selective laser melting process, as previously described [9]. Following the 3D printing process, the implants were cleaned successively in acetone, 96% ethanol, and demineralized water (each step for 5 min under ultrasonication) and were then blow-dried at room temperature.

The PEO process was performed using a research setup equipped with an AC power supply (50 Hz, type ACS 1500, ET Power System Ltd., Eyam, UK), a data acquisition system (SCXI, National Instruments, Austin, TX, USA), and a thermostatic bath (Thermo Haake, Karlsruhe, Germany) [9]. The implants were used as anode materials in the double-jacket electrolytic cell. The cathode was a cylindrical part made from stainless steel. The electrolyte solution consisted of an aqueous solution containing 0.15 M calcium acetate (Sigma-Aldrich, Zwijndrecht, The Netherlands) and 0.02 M calcium glycerophosphate (ISALTIS, Lyon, France). The nanoparticles (AgNPs 0.5 g L^−1^, rGO 0.5 g L^−1^, and rGO+Ag 1:1 mixtures of 0.2, 1.0, 2.0, and 3.0 g L^−1^) were ultrasonicated in demineralized water for 10 min and subsequently added to the electrolytes while stirring for 5 min. A constant electrolyte temperature of 5 ± 2 °C was ensured throughout the PEO process. The PEO treatment was performed at a constant current density of 20 A dm^−2^ for 5 min. The voltage–time (V–t) curves were recorded during the treatment. After the PEO process, the implants were firstly rinsed with tap water for 5 min and then with demineralized water for 1 min to ensure that residual electrolytes and nanoparticles were removed. All the experimental groups are presented in Table 1.

### 2.3. Characterization of Nanoparticles and Implants

#### 2.3.1. Raman Spectroscopy

The chemical bonding state of the rGO powder was studied using a LabRAM HR confocal Raman microscope (Horiba, Kyoto, Japan) equipped with an argon ion laser, which was operated at 514 nm and had a spectral resolution of ~0.3 cm^−1^. The Raman spectra were collected from 1000 to 3000 cm^−1^.

#### 2.3.2. X-ray Diffraction (XRD)

A Bruker D8 advanced X-ray diffractometer (Bruker, Billerica, MA, USA) with Bragg–Brentano geometry bearing a Lynxeye position sensitive detector was used to analyze the crystalline phase composition of rGO particles and implants. The CuK_α_ radiation detector worked at 45 kV and 40 mA. The data were captured within a 2θ range of 20–120° using a step size of 0.034° and a counting speed of 10 s step^−1^, and were then identified using the Bruker DiffracSuite.Eva 4.1 software (Bruker, Billerica, MA, USA).

#### 2.3.3. Scanning Electron Microscopy (SEM)

The morphology and elemental analysis of the rGO and rGO+Ag nanoparticles as well as of the implants were assessed using a JSM-IT100LA scanning electron microscope (JEOL, Tokyo, Japan) and a Hitachi S-3000N scanning electron microscope (Hitachi, Tokyo, Japan), both supplied with an energy-dispersive X-ray spectrometer (EDS). Prior to imaging, the samples were secured on a double-sided carbon tape and exposed to 18 s of gold sputtering to enhance their electrical conductivity.

#### 2.3.4. Particle Size and Zeta Potential

The size, as well as the surface charge (zeta potential) of the particles (rGO, Ag, and rGO+Ag) in the electrolyte, were evaluated with a Zetasizer Nano ZS coupled with an MPT-2 Titrator (Malvern Panalytical, Malvern, UK). Disposable cuvettes (DTS1070, Malvern Panalytical, Malvern, UK) were used, and the measurements were taken at room temperature. The measurements were performed at least 3 times.

#### 2.3.5. Atomic Force Microscopy (AFM)

The local topography of the solid implants was analyzed using a Dimension Edge™ atomic force microscope (Bruker, Camarillo, CA, USA) supplemented with Nanodrive v8.05 software. An antimony (n) doped silicon tip was used in the tapping mode. The pixel resolution and scan rate were set at 256 × 256 and 0.6 Hz, respectively. The samples were prepared by fixing the implants on a double-sided carbon tape. The scanning probe data were analyzed using Gwyddion software (Czech Metrology Institute, Jihlava, Czech Republic).

#### 2.3.6. Inductively Coupled Plasma Optical Emission Spectrometry (ICP-OES)

A PerkinElmer Optima 3000DV analyzer (PerkinElmer, Zaventem, Belgium) was used to monitor the release kinetics of Ag^+^. Implants (*n* = 3 per group) were cut into samples of 1.5 cm lengths. Dark-brown Eppendorf tubes were used to contain the samples in 1 mL of phosphate-buffered saline (PBS), which were then incubated inside a thermostatic water bath (JUBALO SW22, JUBALO GmbH, Seelbach, Germany) at 37 °C. On days 1, 2, 4, 7, 14, and 28, the solution was collected for the measurements and was replaced by fresh liquid.

### 2.4. Antibacterial Assays

#### 2.4.1. Electron Paramagnetic Resonance (EPR)

The free radicals generated on the implant surfaces were analyzed by EPR spin trapping using an EMXplus X-band EPR spectrometer (Bruker BioSpin, Leiderdorp, The Netherlands) and the spin trap 5,5-dimethyl-pyrroline N-oxide (DMPO). For most ROS, direct detection cannot be realized by EPR due to the short lifetimes [46]. The lifetimes of hydroxyl (·OH), superoxide anion (·O_2_^−^), hydrogen peroxide, and nitric oxide (NO_x_) radicals were reported as ~2.7, 1.3, 1.4, and 1.2 μs, respectively [47]. However, a relatively stable free radical (spin adduct), which is detectable by EPR spectroscopy, can be easily formed as a result of the reaction of diamagnetic nitrone spin traps and ROS [48]. Herein, DMPO was applied as a spin trap to capture the ROS generated by the different implants.

The implants (*n* = 3 per group) were inserted inside quartz capillary tubes, which had an internal diameter of 1 mm, and 10 µL of PBS containing 20 mM DMPO was added. The measurements were conducted at room temperature without any direct illumination. Spectra were obtained using a microwave source with a power of 20 mW and a frequency of 9.79 GHz. The modulation frequency was set at 100 kHz and the modulation amplitude was 1.0 Gauss. Prior to measuring the free radical formation in PBS, an EPR spectrum of the implants without solution was measured, which served as the baseline, since a relatively broad V^4+^ signal was present in the PEO-treated implant. Time-dependent EPR spectra were recorded for 3 h. After the measurements, baseline correction was performed, followed by data simulation using the SpinFit module of the Xenon software (Bruker BioSpin, Leiderdorp, The Netherlands).

#### 2.4.2. Zone of Inhibition

In order to explore the in vitro leachable antibacterial activity of the implants bearing rGO, AgNPs, and rGO+Ag, the agar diffusion method was applied with methicillin-resistant *Staphylococcus aureus* (MRSA) USA300 [49]. The untreated AM implants and the PEO-treated implants without any nanoparticles were also tested and served as the reference groups. A single colony of MRSA was suspended in 3 mL tryptic soy broth (TSB, Sigma-Aldrich, Zwijndrecht, The Netherlands). The inoculum was subsequently cultured for 3 h at 37 °C under constant shaking. The bacterial concentration was determined by measuring the optical density at 600 nm (OD_600nm_). The culture was then diluted to OD_600nm_ 0.01 (~10^7^ colony forming units mL^−1^) in TSB. The bacterial suspensions were plated with a sterile cotton swab. The implants (1.5 cm in length) were placed on the plates and were incubated for 24 h at 37 °C. Subsequently, the inhibition zones were determined with ImageJ image processing software (LOCI, University of Wisconsin, Madison, WI, USA) by measuring the areas of the zones of inhibition (*n* = 3 per group).

### 2.5. Cell Response to the Implants

MC3T3-E1 preosteoblast cells (Sigma-Aldrich, Zwijndrecht, The Netherlands) were first pre-cultured until 80–90% confluency in α-Minimum Essential Medium (α-MEM without ascorbic acid, Thermo Fisher Scientific, Landsmeer, The Netherlands) supplemented with 1% penicillin–streptomycin and 10% fetal bovine serum (both from Thermo Fisher Scientific, Landsmeer, The Netherlands) at 37 °C and 5% CO_2_. The implants were then cut into specimens of 1 cm length and sterilized by autoclaving at 121 °C for 20 min. For cell seeding, each specimen was placed in a 0.2 mL tube with a suspension of 1 × 10^5^ cells in 150 µL α-MEM. The samples were incubated at 37 °C and 5% CO_2_ and flipped every 20 min during the first 2 h to ensure the homogeneous seeding of the 3D volume-porous implants. Following seeding, the implants were placed in 48-well plates with 200 µL fresh α-MEM/well. After 48 h of incubation, the α-MEM was replaced by a differentiation medium consisting of α-MEM supplemented with 50 μg mL^−1^ ascorbic acid and 4 mM β-glycerophosphate (both from Sigma-Aldrich, Zwijndrecht, The Netherlands). The culture medium was refreshed every 2–3 days.

#### 2.5.1. Metabolic Activity

The metabolic activity of the preosteoblast cells on days 1, 4, 7, and 11 was investigated using the PrestoBlue assay (Thermo Fisher Scientific, Landsmeer, The Netherlands). On each of the days selected for analysis, the implants (*n* = 4 per group) were transferred to a new 48-well plate and incubated in α-MEM supplemented with 10% vol. PrestoBlue reagent. After 1 h of incubation at 37 °C and 5% CO_2_, aliquots of 100 µL supernatant from each sample were transferred into a 96-well plate and analyzed by measuring absorbance using a microplate reader (Victor X3, PerkinElmer, Nederland B.V., Groningen, The Netherlands). The entire set of experiments (i.e., *n* = 4 per group) was performed one more time to ensure data reproducibility.

#### 2.5.2. Cell Morphology

The morphologies of the cells adhered on the surfaces of the implants were observed by SEM after 1, 7, and 11 days of culture. The implants (*n* = 2 per group) were rinsed in PBS (Thermo Fisher Scientific, Landsmeer, The Netherlands) and were fixated with 4% formaldehyde/1% glutaraldehyde (Sigma-Aldrich, Zwijndrecht, The Netherlands) for 10 min. After fixation, the implants were rinsed twice with demineralized water for 5 min, dehydrated in 50% ethanol for 15 min and in 70% and 96% ethanol (each 20 min), then air-dried for 2 h. Before SEM imaging, a thin gold layer was sputtered on the specimens.

#### 2.5.3. Alkaline Phosphatase Activity

Osteogenic differentiation was examined by evaluating the alkaline phosphatase (ALP) activity of the cells after 7 and 11 days. The implants (*n* = 4 per group) were rinsed in PBS and inserted in 250 µL 0.1% Triton/PBS (Sigma-Aldrich, Zwijndrecht, The Netherlands). The cells were detached from the implants by ultrasonication for 10 min and were then incubated with 20 mM p-nitrophenyl phosphate disodium salt (pNPP) in diethanolamine buffer (both from Sigma-Aldrich, Zwijndrecht, The Netherlands) for 10 min at 37 °C. Subsequently, 250 µL of 0.1 M NaOH was added to terminate the reaction. The absorbance was then measured at 405 nm by using the same Victor X3 microplate reader. The ALP activity was normalized to the total protein levels determined using the BCA protein assay kit (Invitrogen, Thermo Fisher Scientific, Landsmeer, The Netherlands). The entire experiment (i.e., *n* = 4 per group) was performed twice to ensure data reproducibility.

### 2.6. Statistical Analysis

The results are represented as means ± standard deviations. Statistical analyses were performed using one-way ANOVA (GraphPad Software, La Jolla, CA, USA). *: *p* < 0.05, **: *p* < 0.01, ***: *p* < 0.001.

## 3. Results

### 3.1. Physicochemical Properties of the Nanoparticles

**Figure 1:**
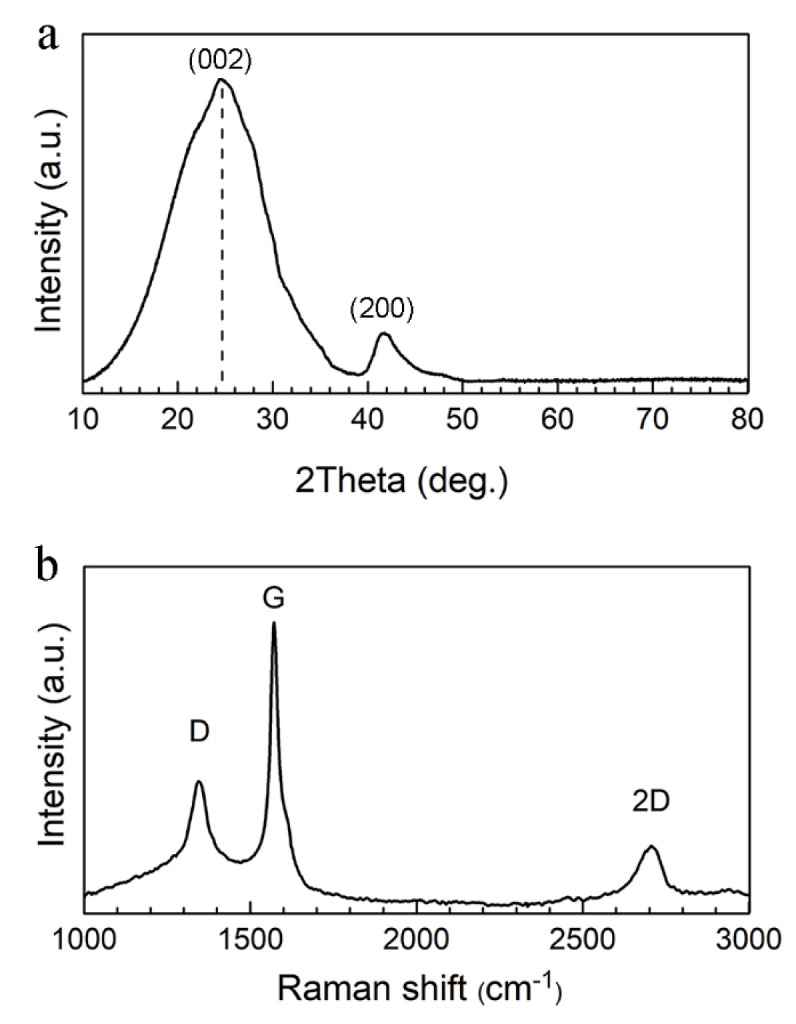
Structural characterization of the rGO nanosheets: (**a**) XRD analysis and (**b**) Raman spectra. (The G band arose from the stretching of the C−C bond. The D band is due to the disordered structure of rGO. The 2D band was attributed to the splitting of electron bands.)

The XRD patterns of the rGO particles exhibited two peaks (Figure 1a). The broad reflection peak (002) at 24.3° suggests the presence of a few layers of rGO sheets with an interlayer distance of about 3.67 Å. For crystalline graphite, this value is 3.40 Å. The increase in lattice distance resulted from the oxygen-containing functional groups on rGO [50]. The small peak at 42.0° was attributed to the (200) plane of the rGO sheets.

The Raman spectra of the rGO particles (Figure 1b) indicated the G band at around 1570 cm^−1^ as a result of the in-plane vibrations of sp^2^ bonded carbon atoms, which is also a characteristic feature of the graphitic layers. The D band at around 1350 cm^−1^ is due to the out-of-plane vibrations attributed to structural defects, indicating a defective graphene structure resulting from the redox reaction during the preparation. The intensity ratio of the D and G bands (I_D_/I_G_ = ~0.56) suggested a low degree of oxidation [51]. Furthermore, the noticeable 2D band (around 2700 cm^−1^) shows that the particles were not a monolayer of rGO but had a multi-layer structure.

**Figure 2:**
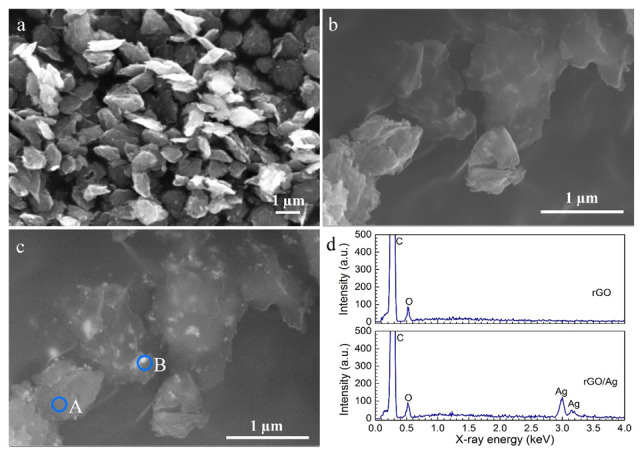
SEM images of (**a**) the rGO nanosheets after ultrasonication in isopropanol, (**b**) the rGO+Ag mixture (1:1), (**c**) the same image as in (**b**) but in the back-scattered mode, and (**d**) the EDS analysis of areas A (rGO nanosheets) and B (rGO+Ag mixture), as indicated in (**c**).

The morphology of the rGO sheets (Figure 2a) exhibited small sheets (≤1.0 μm in size) consisting of a few rGO monolayers, which indicates that the material was micro-mechanically exfoliated by ultrasonication. Sharp edges were also visible, indicating that the rGO sheet thickness was in the nanoscale range. Comparing the SEM image and the backscattered micrograph of the same spot, the rGO sheets were less bright than AgNPs (Figure 2b,c). A number of small AgNPs (<50 nm) adhered to the rGO sheets and were mostly distributed on the edges of the sheets. Some clusters of AgNPs up to 200 nm were observed on several rGO sheets. Moreover, the combination of AgNPs and rGO was also confirmed by EDS analysis (Figure 2d). The C peak and the weak peak of oxygen indicate the presence of rGO, whereas the additional Ag peak demonstrates the mixture of rGO and AgNPs (spots A and B as indicated in Figure 2c).

**Figure 3:**
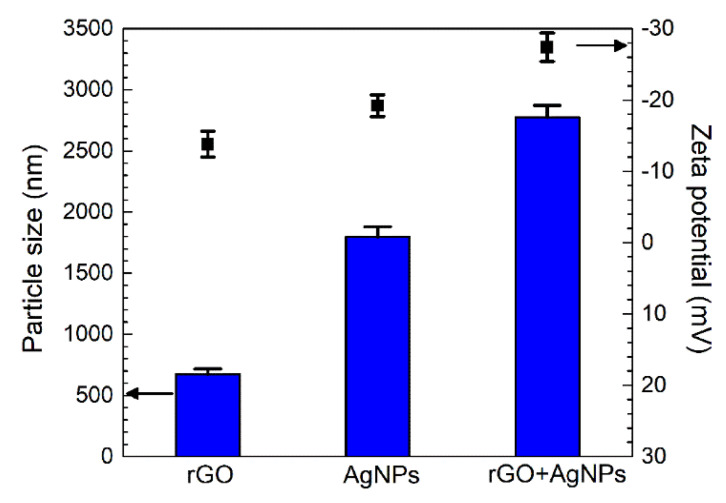
Average particle sizes and zeta potentials of the 0.5 g L^−1^ rGO nanosheets, 0.5 g L^−1^ AgNPs, and 0.5 g L^−1^ rGO + 0.5 g L^−1^ Ag mixture in the PEO electrolytes.

The average size of rGO sheets was measured to be approximately 673 ± 82 nm (Figure 3), which is consistent with their size displayed in Figure 2a. The AgNPs tended to cluster in the PEO electrolytes, forming larger aggregates of about 1797 ± 82 nm despite their higher zeta potential (−19.2 ± 1.5 mV) as compared to the rGO particles (−13.8 ± 1.8 mV). Moreover, the size of the rGO+Ag nanoparticles was larger than those of the rGO and Ag nanoparticles. The combination of rGO with AgNPs seems to double the zeta potential of the resulting mixture (−27.4 ± 2 mV) compared to single rGO nanosheets.

### 3.2. Synthesis of Antibacterial Implants

#### 3.2.1. PEO Process

**Figure 4:**
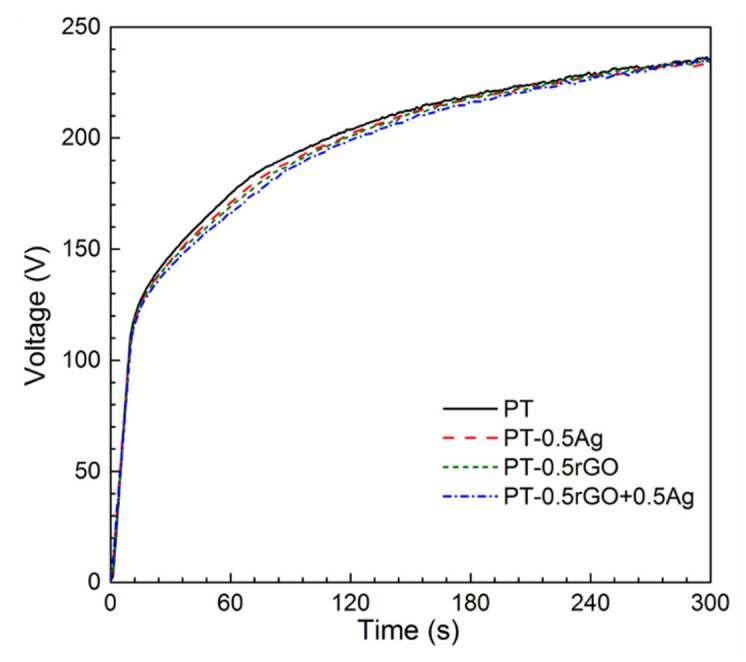
The V–t curves recorded during the PEO process of the PT, PT-0.5Ag, PT-0.5rGO, and PT-0.5rGO+0.5Ag implants.

The V–t curves for all the samples exhibited similar characteristics (Figure 4), indicating that the incorporation of the nanoparticles had no significant effects on the growth process of the PEO layer. The rapid voltage increase at the beginning of the PEO treatment is related to the formation of a dense barrier oxide layer. The growth rate was calculated to be around 12.93 V s^−1^. After the dielectric breakdown occurred (circa 10 s), the growth rate slowed down to 0.88 V s^−1^, indicating the gradual development of a porous oxide layer.

#### 3.2.2. Phase Composition of the Implants

**Figure 5:**
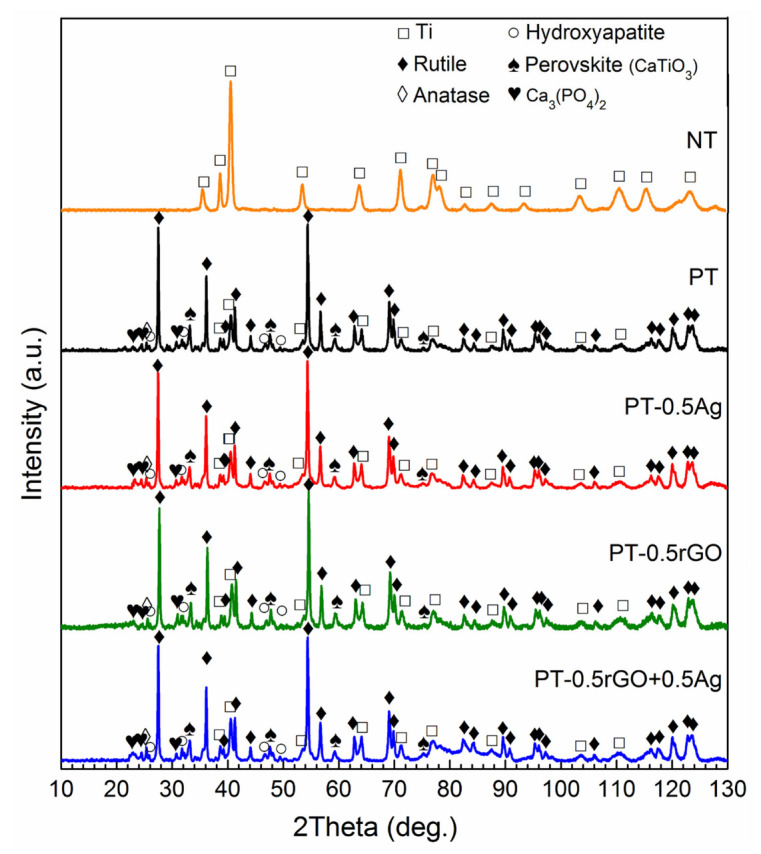
XRD patterns of the NT, PT, PT-0.5Ag, PT-0.5rGO, and PT-0.5rGO+0.5Ag implants.

The XRD analysis (Figure 5) showed no difference in phase composition between the implants prepared with and without particles. The non-treated AM implants (NTs) were also measured as a reference. The implants exhibited the diffraction peaks of the titanium substrate and the crystalline titanium dioxide peaks after PEO. The oxide layer consisted mainly of rutile, according to the considerably high intensity of the diffraction peaks. Moreover, the presence of some low diffraction peaks of anatase, perovskite (CaTiO_3_), calcium phosphate (Ca_3_(PO_4_)_2_), and hydroxyapatite (Ca_10_(PO_4_)_6_(OH)_2_) were detected in the XRD patterns as well. However, the XRD peaks of rGO and Ag did not appear in the XRD patterns of the implants with nanoparticles.

#### 3.2.3. Morphology and Composition of Implants

**Figure 6:**
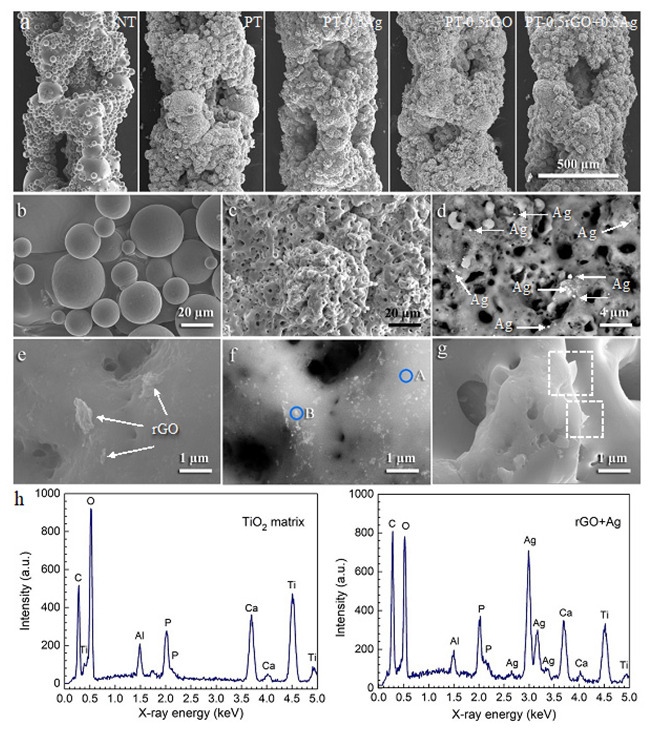
(**a**) Low-magnification SEM micrographs of the NT, PT, PT-0.5Ag, PT-0.5rGO, and PT-0.5rGO+0.5Ag implants. High-magnification SEM micrographs of (**b**) NT and (**c**) PT implants. (**d**) Back-scattered image of the PT-0.5Ag implant. SEM micrograph (**e**) and back-scattered image (**f**) of the PT-0.5rGO+0.5Ag implant. (**g**) ‘Nano-knives’ area of the PT-0.5rGO+0.5Ag implant. (**h**) EDS spectra of areas of the TiO_2_ matrix (marked as A in (**f**)) and rGO+Ag nanoparticles (marked as B in (**f**)) of the PT-0.5rGO+0.5Ag implant.

The SEM images clearly exhibited that the as-printed specimens possessed interconnected macro-pores formed by molten spherical-shaped titanium alloy particles with numerous unmolten or partially molten smooth spheres adhered to them (Figure 6a). A local rough surface could be observed on the oxidized implants, but there was no significant difference in the morphology at a low magnification between the implants with and without nanoparticles. The smooth surface of the NT implant changed into a rugged topography after the PEO (Figure 6b,c) due to the formation of micro- and nanopores.

The incorporation of rGO nanosheets and AgNPs could be identified at high magnifications. The back-scattered images (Figure 6d) showed that the bright spots representing AgNPs with a wide range of size from several nanometers to several hundred nanometers were randomly distributed on the surface of the PT-0.5Ag specimens. Reduced GO nanosheets with a size of <1 µm were observed on the PT-0.5rGO+0.5Ag specimens (Figure 6e). Nano-sized Ag particles were incorporated on the surface of the implants while being attached to the rGO nanosheets (Figure 6f). The AgNPs adhered more to the edges of the nanosheets than to their surfaces. Some rGO nanosheets were embedded perpendicularly to the implant surface (e.g., the PT-0.5rGO+0.5Ag specimen in Figure 6g), forming ‘nano-knife’ structures. EDS analysis evidenced the co-existence of Ag and C in the areas of embedded AgNPs and rGO sheets, whereas no Ag or C peaks were detected in the TiO_2_ matrix (Figure 6h). Furthermore, the elements of the matrix, such as Ti, O, and Al, were identified along with the PEO electrolytic constituents, such as Ca and P.

**Figure 7:**
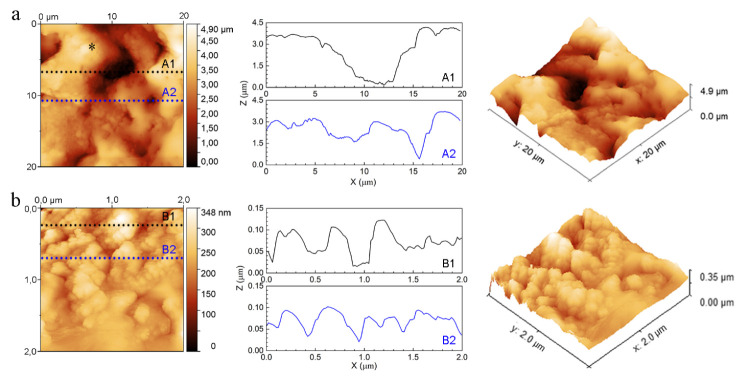
Two- and three-dimensional AFM surface micrographs together with the line analysis (marked in the 2D graphs as A1, A2, and B1, B2) of the PT-0.5rGO+0.5Ag implant: (**a**) large scanning area of 20 × 20 µm^2^ and (**b**) small scanning area of 2 × 2 µm^2^ (see * in (**a**)).

AFM analysis was used to investigate the local topography of the implants bearing the rGO+Ag nanoparticles. Instead of porous AM implants, solid titanium implants oxidized under the same preparation conditions were used because the porous AM implants were not suitable for AFM analysis due to their 3D porous geometry. The representative topographies of 20 × 20 µm^2^ areas (Figure 7a) together with very local topographies of 2 × 2 µm^2^ areas (Figure 7b) were acquired by AFM. Line profiles, labeled in the 2D images, were illustrated as well. Rough surfaces with pores in a wide diameter range and a maximum height of 4.902 µm were observed (Figure 7a). The sizes of the pores were about 2.0 to 9.2 µm in diameter. Unlike the relatively smooth morphology observed by SEM in Figure 6e,g, the AFM analysis showed a very fine submicron-scale roughness in these areas (Figure 7a, 2D, marked area). The two- and three-dimensional images in Figure 7b exhibited a rough surface with a maximum height of 0.348 µm. Large protrusions (~0.3–0.6 µm) with small particles (<0.1 µm) on their surface were observed from the line profile. The mean roughness (*S_a_*) of both areas was also analyzed, and the values were around 714 nm and 30 nm for the 20 × 20 µm^2^ and 2 × 2 µm^2^ areas, respectively.

### 3.3. Antibacterial Properties of the Biofunctionalized Implants

#### 3.3.1. Ag Release Kinetics

**Figure 8:**
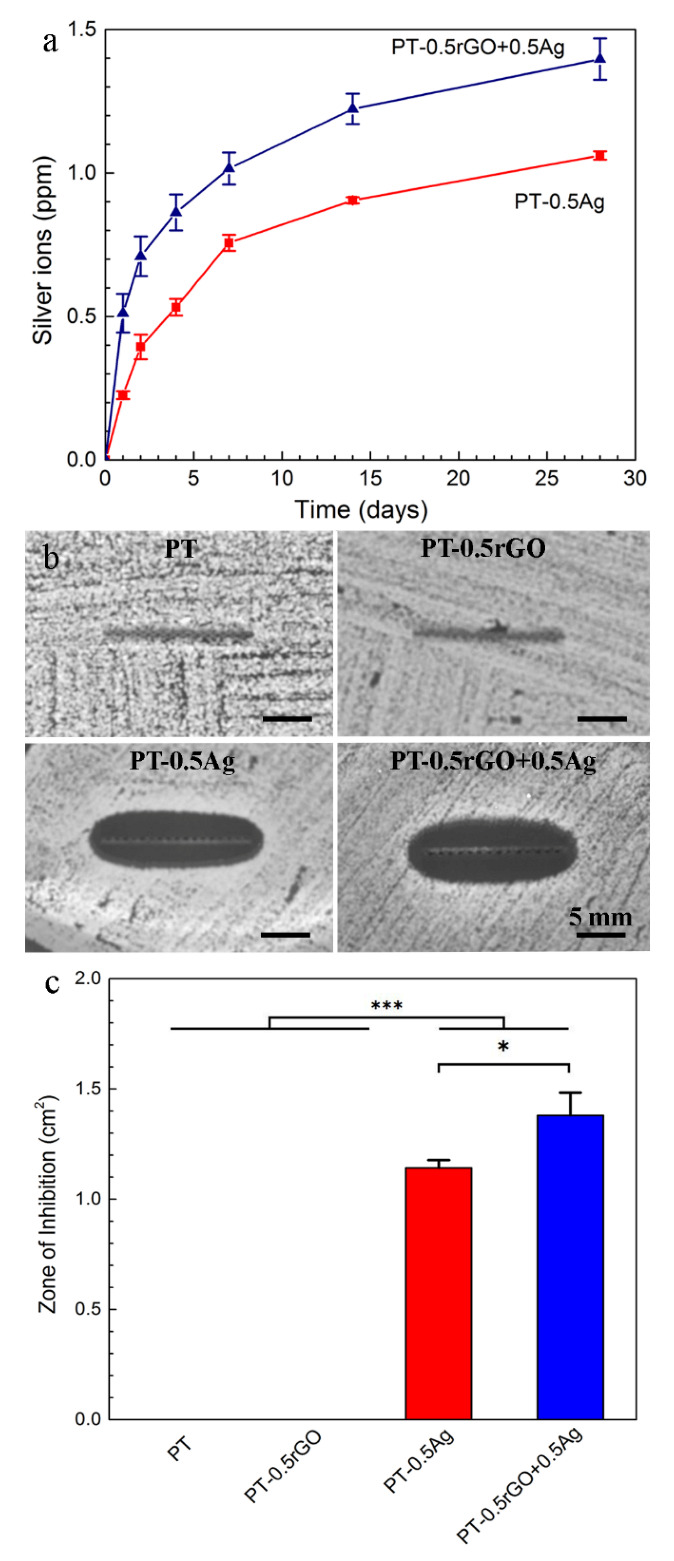
(**a**) Silver release (*n* = 3) of the PT-0.5Ag and PT-0.5rGO+0.5Ag implants during 28 days of immersion in PBS. (**b**) Inhibition zone images of the PT, PT-0.5Ag, PT-0.5rGO, and PT-0.5rGO+0.5Ag implants against MRSA after 1 day. (**c**) Inhibition areas of the PT, PT-0.5Ag, PT-0.5rGO, and PT-0.5rGO+0.5Ag implants calculated from (**b**) (*n* = 3). *: *p* < 0.05, ***: *p* < 0.001.

Both implants gradually released Ag^+^ for up to 28 days (Figure 8a). The cumulative release rate was high during the first 4 days, then it decreased gradually with immersion time. After 28 days, the concentration of silver ions in the PBS was 1.06 ± 0.03 ppm for the PT-0.5Ag group, but it increased by about 32% in the presence of rGO (1.40 ± 0.01 ppm) nanoparticles. With the addition of rGO in the PEO electrolytes, the rate of Ag^+^ release and the total release concentration during the 28 days of immersion were enhanced.

#### 3.3.2. Zone of Inhibition

No zone of inhibition was observed around the PT and PT-0.5rGO implants (Figure 8b). The inhibition zone of the PT-0.5rGO+0.5Ag specimens against MRSA (Figure 8c) was determined as 1.380 ± 0.137 cm^2^, which was significantly larger than that of the PT-0.5Ag implants (1.175 ± 0.047 cm^2^; *p* < 0.05).

#### 3.3.3. Detection of ROS Generation

**Figure 9:**
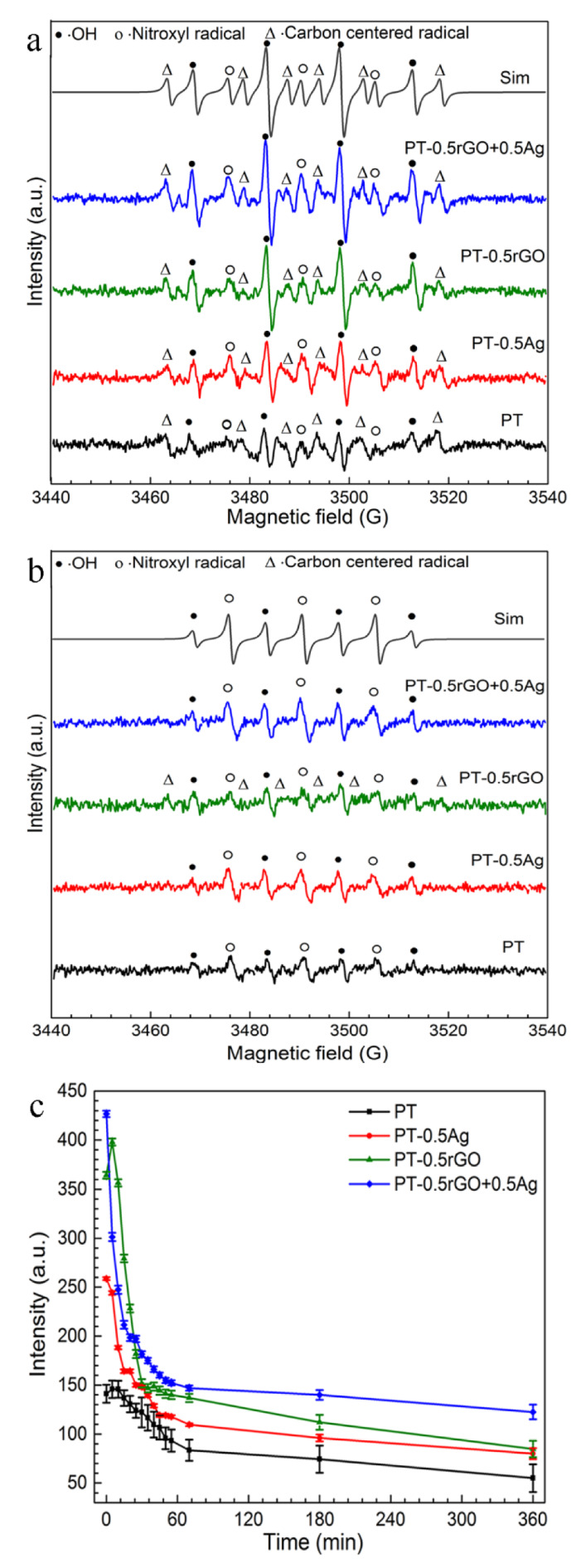
EPR spectra of the PT, PT-0.5Ag, PT-0.5rGO, and PT-0.5rGO+0.5Ag implants: (**a**) after 5 min and (**b**) after 3 h. Simulated (Sim) curves of the PT-0.5rGO+0.5Ag EPR spectra were also presented above. (**c**) Time series of DMPO/·OH signal amplitudes during 3 h measurement (*n* = 3).

The DMPO/·OH adduct, identified by a characteristic four-peak spectrum with relative magnitudes of 1:2:2:1, was observed at varying intensities for all the specimens (Figure 9a) [52]. Furthermore, the typical six-peak spectrum of a DMPO/∙Carbon centered radical was observed. This signal can be attributed to methyl, hydroxymethyl, other carbon-centered radical adducts, or a combination thereof, which is commonly seen in DMPO spin trap experiments [53,54]. In this case, the organic radical may come from the carry-over of calcium acetate and calcium glycerophosphate from the PEO treatment. The spectrum also demonstrated additional lines, which consisted of a 1:1:1 triplet. Some previous studies have also reported this kind of signal and named it either nitroxide-like radical [55,56] or nitroxyl radical (·NO_x_) [57], which probably resulted from the degradation of the DMPO/·OH adduct by C-N bond cleaving and pyrroline ring opening [56,58,59,60]. However, no agreement was reached on the structure or mechanism [61]. Accordingly, the EPR data were simulated using the characteristic hyperfine interactions of the three types of DMPO adducts, based on their hyperfine splitting parameters: the DMPO/·OH with α_N_ = α_H_^β^ = 14.7 G, the DMPO/nitroxide-like radical with α_N_ = 14 G, and a DMPO/∙Carbon centered radical with α_N_ = 16.4 G and α_H_^β^ = 23.4 G. The g value for each signal was 2.0066 ± 0.0002. The EPR parameters are consistent with the reported values for the radicals in DMPO, and the simulated signal fitted well with the observed spectra [53,59,61,62].

The same radical adducts were observed for the implants with and without particles at the beginning of the measurement (Figure 9a). The EPR signal intensity was higher for the implants oxidized in the presence of particles, and the PT-0.5rGO group showed a higher intensity in the DMPO/·OH peaks than the PT-0.5Ag group. Additionally, the addition of rGO particles induced a significant increase in the DMPO/·OH signal. After 3 h of measurement, the amplitudes of the EPR signals decreased significantly (Figure 9b). The peaks indicating the DMPO/∙Carbon centered radical disappeared, except in the spectra of the PT-0.5rGO implants. Only two adducts, DMPO/·OH and DMPO/nitroxide-like radical, were observed in the EPR spectra after 3 h.

The spectra were simulated and the double integrals of the radicals were obtained. The time series of the DMPO/·OH area in 3 h exhibited a rapid decrease (Figure 9c). Compared with the PT specimens, the AgNP-incorporated specimens induced a significant increase in the ·OH spin adduct at the beginning of the measurement. The signal area was even higher for the implants with the rGO particles (PT-0.5rGO and PT-0.5rGO+0.5Ag). A significant decrease in the area was observed in the EPR spectrum of the implants with particles during the first 30 min as compared with the PT implants, and decayed slowly afterwards. The spectra were relatively stable after 70 h, and the areas of the PT-0.5rGO+0.5Ag implants were still greater than the PT-0.5rGO and PT-0.5Ag specimens until the end of the measurements.

### 3.4. In Vitro Cell Response

**Figure 10:**
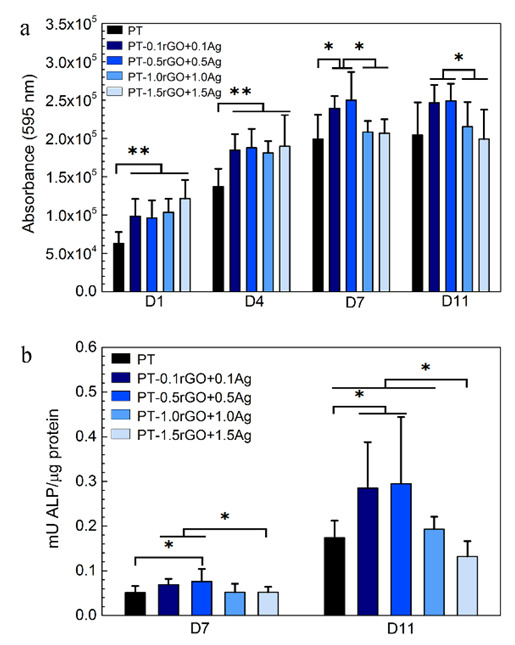
Metabolic activity (**a**) and ALP activity (**b**) of MC3T3-E1 cells cultured on the PT, PT-0.5Ag, PT-0.5rGO, PT-0.5rGO+0.5Ag, PT-1.0rGO+1.0Ag, and PT-1.5rGO+1.5Ag implants (*n* = 8). *: *p* < 0.05, **: *p* < 0.01.

The general trend of the PrestoBlue results (Figure 10a) showed an increase in the metabolic activity of the preosteoblast cells on all the implants during the 7 days of culture, followed by a steady state after that. Moreover, a closer look at the data suggested that all the PT-rGO+Ag implants led to higher metabolic activities after 1 and 4 days of culture relative to the PT control. At the later time points, the PT-0.1rGO+0.1Ag and PT-0.5rGO+0.5Ag specimens showed higher activity levels than the other loaded implants. The metabolic activity detected for these implants was either more elevated than the PT control (after 7 days) or comparable to the PT control (after 11 days).

The measurements of ALP activity (Figure 10b) indicated that all the groups enhanced the osteogenic differentiation of the mouse preosteoblast cells, although to different extents. The PT-0.1rGO+0.1Ag and PT-0.5rGO+0.5Ag surfaces stimulated osteogenic differentiation compared to the other loaded implants, as indicated by the significantly higher levels of ALP activities measured for these implants after 7 and 11 days of culture. The cells cultured on the PT-0.5rGO+0.5Ag implants showed the most increased ALP activity at both time points.

**Figure 11:**
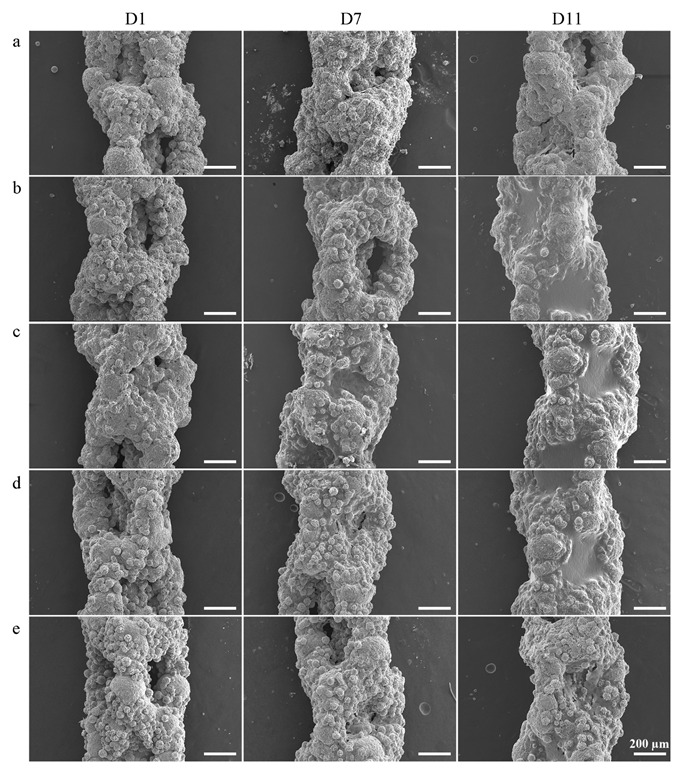
SEM images of the implants after 1, 7, and 11 days of cell culture at low magnification: (**a**) PT, (**b**) PT-0.1rGO+0.1Ag, (**c**) PT-0.5rGO+0.5Ag, (**d**) PT-1.0rGO+1.0Ag, and (**e**) PT-1.5rGO+1.5Ag.

**Figure 12:**
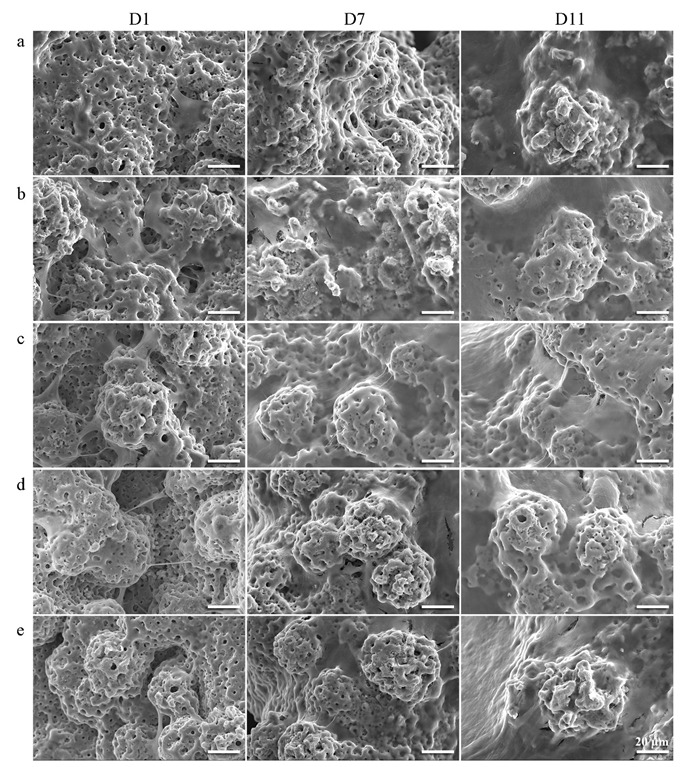
SEM images of the implants after 1, 7 and 11 days of cell culture at high magnification: (**a**) PT, (**b**) PT-0.1rGO+0.1Ag, (**c**) PT-0.5rGO+0.5Ag, (**d**) PT-1.0rGO+1.0Ag, and (**e**) PT-1.5rGO+1.5Ag.

The preosteoblasts attached and stretched on the rough and porous implants after 1 day of culture were able to bridge some of the gaps between the oxidized particles protruding from the surfaces (Figure 11 and Figure 12). No significant differences between the implants from different groups were observed (Figure 11a–e). However, the cells seemed to have already formed a network (Figure 12a–e) which was well integrated with the surfaces on the PT-0.1rGO+0.1Ag and PT-0.5rGO+0.5Ag implants (Figure 12b,c). After 7 days of incubation, cells proliferated and started to fill the large pores (~500 µm in diameter) of the 3D-printed implants (Figure 11a–e). Interestingly, the best coverage was observed for the PT-0.5rGO+0.5Ag implants (Figure 11c). After 11 days, all the implants were fully covered except for the PT (Figure 11a) and PT-1.5rGO+1.5Ag (Figure 11e) groups.

## 4. Discussion

Reduced GO is a promising multifunctional bioactive material because of its unique structural and physicochemical properties. The large surface area and particle adsorption capacity make it an ideal support for the dispersion and stabilization of metallic nanomaterials. However, its antibacterial effects and potential cytotoxicity are primarily determined by the application state [16]. In this study, we demonstrated for the first time the feasibility of using PEO to incorporate rGO+Ag nanoparticulate materials onto the surfaces of porous, geometrically complex, 3D-printed titanium implants. The implants clearly showed enhanced antibacterial activity against MRSA compared to the implants bearing single AgNPs as an antibacterial agent while also offering enhanced cell proliferation and osteogenic differentiation of preosteoblasts. Additionally, the concentration of AgNPs on the implant surfaces and the Ag^+^ released over time increased in the presence of rGO. Finally, the ‘nano-knife’ structure of rGO sheets around the PEO pores and the more persistent generation of ROS exhibited further antibacterial potential. Altogether, the presented results show the multifunctional potential of the AM volume-porous implants developed here.

### 4.1. rGO+Ag Preparation and Dispersion

AgNPs tend to aggregate and thus increase their size, especially in high-salt solutions, such as the PEO electrolyte solution used in this study. The addition of rGO nanosheets provided a better dispersion of AgNPs. As provided by the supplier, the AgNPs had diameters between 7 and 25 nm. When added to the PEO electrolytes, AgNPs aggregated, and the average size increased significantly to 1797 ± 82 nm (Figure 3). This may reduce the total surface area of the AgNPs to which the bacteria can be exposed, hence reducing the antibacterial properties of the obtained implants [63,64,65]. The rGO nanosheets have a flat (2D) morphology (Figure 2b), while the size analyzer can only regard the particles as spheres [24]. Therefore, the obtained size of rGO nanosheets represented the lateral dimension of the nanosheets, which was still in the provided size range, between 500 and 800 nm. The rGO+Ag mixtures were formed after ultrasonication. Small AgNPs were primarily attached at the edges of the rGO nanosheets (Figure 2b,c) due to the absorption of the oxygen-containing functional groups at the edges. However, AgNP clusters with relatively large diameters (~100 nm) can also be captured by several rGO monolayers due to the high adsorption capacity resulting from the large surface area of the rGO [31,66].

### 4.2. rGO Nanosheets Enhance upon AgNP Incorporation during PEO

The rGO+Ag nanoparticles had a zeta potential of −27.4 ± 2.0 mV, indicating an improved dispersion stability compared to AgNPs (−19.2 ± 1.5 mV, Figure 3). During the PEO treatment, no significant changes were observed in the V–t curves between the specimens from different groups (Figure 4). Moreover, the presence of the particles did not change the phase composition (Figure 5) of the obtained PEO layers. The agglomerated Ag particles with a diameter of >100 nm, which were randomly distributed on the PT-0.5Ag sample, could not be found on the PT-0.5rGO+0.5Ag implant surface (Figure 6d,f). It seems that with the addition of the rGO nanosheets, the dispersion of AgNPs on the surfaces of the implants as well as on the incorporated rGO nanosheets was improved, indicating an improvement in the distribution of incorporated AgNPs. Furthermore, after immersion in PBS for 28 days, the concentration of Ag^+^ released from the PT-0.5rGO+0.5Ag implants was about 31.6% higher than that of the PT-0.5Ag implants (Figure 8a). This supports the idea that the presence of rGO nanosheets could increase the incorporation efficiency of AgNPs.

### 4.3. Antibacterial Activity

The antibacterial activities of nanoparticles can be ascribed to many factors, including penetration of the cell wall, ROS generation, physical piercing, and toxic metal ion release [57,67]. However, the antibacterial properties of nanoparticles vary significantly after their incorporation on the surfaces of implants due to loss of mobility. Therefore, EPR spin trapping, an established method to identify and quantify the ROS generation mediated by implants, was used in this study. The DMPO/∙OH radical was the major ROS species detected for all the specimens (Figure 9). The ∙OH radical is known to be very reactive, which can lead to oxidative damage and thus result in toxic effects on bacteria. The high intensity of the radical at the initial measurement stage indicated the abundant DMPO/·OH generated by the implants with particles. The intensity for all the implants decreased rapidly in about 20 min, which is consistent with the lifetime of *t*_1/2_ = 23 min reported previously [68,69]. The DMPO/·OH signal persisted till the end of the measurements, even for the implants without particles. This indicates a steady state in the generation and decay of ROS and the potential for antibacterial activities. There have been several reports on the antibacterial capacities of TiO_2_ nanoparticles [70,71,72]. Therefore, crystallized TiO_2_, the main structural phase of the PEO layer, may be a contributor to ROS generation.

At the beginning of the measurements, the implants from the PT-0.5Ag, PT-0.5rGO, and PT-0.5rGO+0.5Ag groups, respectively, increased the DMPO/∙OH radical area by 86%, 186%, and 204% as compared to the control group (PT). Accordingly, rGO more strongly induced the generation of ∙OH than AgNPs when incorporated on the surface of the PEO layer. The capacity was even higher for the implants with a rGO+Ag mixture than with one type of nanoparticles. After 3 h, the increased DMPO/∙OH areas for the rGO- and AgNP-incorporated implants were approximately equal (46%), whereas that of the PT-0.5rGO+0.5Ag group was still clearly higher (122%). Therefore, the incorporation of rGO+Ag nanoparticles also enhanced the steady-state generation of ROS. The highly improved ability of the implants utilizing the rGO+Ag mixture to continuously mediate ROS generation exhibits their high bactericidal potential.

In addition to ·OH, ∙O_2_^−^ is an effective ROS that could also cause microbial toxicity. Some investigators have reported the formation of ∙O_2_^−^ during AgNP-assisted H_2_O_2_ decomposition [73]. However, no spin adducts attributable to ∙O_2_^−^ were observed in this study. The lifetime of the DMPO/∙O_2_^−^ adduct is only *t*_1/2_ = 45 s [74]. Therefore, it is hard to detect it if it is not continuously generated. There are also other studies reporting that the DMPO/∙O_2_^−^ adduct is unstable and easily decays to a DMPO/∙OH adduct [75]. This also leads to the difficulty in DMPO/∙O_2_^−^ radical detection and may cause misinterpretation of the generation of ∙OH and ∙O_2_^−^. In summary, there are three possible reasons for the absence of ∙O_2_^−^ in this study: (i) no ∙O_2_^−^ was generated, (ii) the generation of ∙O_2_^−^ was not sustainable, and (iii) the fast decay of DMPO/∙O_2_^−^. There are other spin traps that form a more stable superoxide adduct (e.g., BMPO). The BMPO/∙O_2_^−^ adduct has a lifetime of *t*_1/2_ = 23 min [76,77]. Moreover, specific ROS scavengers can be used to determine whether ∙O_2_^−^ is formed. Such specific scavengers include XTT (2,3-Bis (2-methoxy-4-nitro-5-sulfophenyl)-2H-tetrazolium-5-carboxanilide inner salt) [22,78].

The surface microbicidal assay against MRSA showed the leaching antibacterial effect of the implants containing the rGO+Ag mixture. Unlike the ROS results, the NT, PT, and PT-0.5rGO groups did not exhibit any leaching antibacterial effects. However, the implants containing AgNPs displayed certain levels of antibacterial activity (Figure 8b). This may be due to the stability of rGO and TiO_2_ in the medium. The Ag nanoparticles have the ability to release Ag^+^ in body fluids and have been reported to have an antibacterial effect [52,79]. In addition, the inhibition zone around the PT-0.5rGO+0.5Ag implants was significantly larger (up to 29.1%) as compared with the implants from the PT-0.5Ag group (*p* < 0.05). This is consistent with the data for Ag^+^ release after 1 day (Figure 8a). As compared with PT-0.5Ag, the higher Ag^+^ release rate of PT-0.5rGO+0.5Ag and the total concentration of ions after 28 days in PBS suggest an improved long-term antibacterial activity of the implants with the rGO mixture. Furthermore, it is worth noting that the embedded rGO could form a ‘nano-knife’ structure around the pores and thus physically cut bacterial cell walls. Normally, most rGO nanosheets are spread on surfaces in such a way that precludes the orthogonal incision of bacterial cell walls. However, the preferred orientation of the incorporated rGO is affected by the topography of the nanosheets as well as that of the implants. The rGO nanosheets used in this study were sheets with sharp edges (Figure 2b). They have high surface energies due to their large specific surface areas and tend to be adsorbed on implants with flat configurations. However, the cylindrical NT substrate, together with the micro-/nanoporous PEO layer, presented a rough surface. Upon adsorption to this complex surface, the rGO sheets did not have enough flat areas to be attached, leading to the protrusion of the sharp edges from the substrate surfaces. Such sharp edges have a great potential to cause physical damage to bacterial cell walls and complement the effects of ROS generation [19,80]. To date, surfaces with such a ‘nano-knife’ structure have scarcely been reported [81], and the rGO literature is dominated by reports on flat graphene-based coatings or coatings with folds [34,82].

### 4.4. Cell Response

The generation of ROS by nanoparticles benefits antibacterial function on the one hand, while on the other hand it may promote cytotoxicity risks [83,84,85]. Moderate oxidative stress can activate antioxidant reactions, which results in the recovery of redox homeostasis [84]. However, cellular apoptosis and necrosis may happen as a result of the collapse of the defense system under a high concentration of ROS [86]. Multiple conflicting reports about the cytotoxicity of rGO have been published. Some of them suggest that rGO has excellent biocompatibility [87] and promotes the proliferation of L929, MG63 [88,89], and HFOB cells [90,91]. Others have reported some cytotoxic effects of rGO on MC3T3-E1 [92], MCF-7 [84], and A549 [85] and size-dependent toxicity on hMSCs cells [93]. Various factors, including ROS generation, high rGO concentration, large rGO size, and long exposure times have been reported to be detrimental to cell viability [94]. The cytocompatibility of synthetic rGO+Ag mixtures has rarely been studied and presents an even more complex problem in terms of the involved mechanisms.

To address the cytocompatibility of our implants with antibacterial surfaces, we investigated the effects of the rGO+Ag incorporated onto the implant surfaces on the behavior of MC3T3-E1 preosteoblasts, including their metabolic activity, differentiation potential, and morphology, over a period of 11 days. The findings evidenced that the implants loaded with rGO and AgNPs were (highly) cytocompatible, as they supported the adhesion, growth, and differentiation of these cells. However, the effects depended on the concentrations of the rGO+AgNPs mixtures, with the lower concentrations of rGO and AgNPs (i.e., the PT-0.1rGO+0.1Ag and PT-0.5rGO+0.5Ag implants) showing more substantial beneficial effects than the higher concentrations (i.e., PT-1.0rGO+1.0Ag and PT-1.5rGO+1.5Ag). Moreover, these implants stimulated early cell growth (up to day 7) relative to the PT implants, as well as the osteogenic differentiation of cells, as indicated by the higher ALP activity at day 11 as compared to the control group (i.e., PT). These results suggest that the response of mammalian cells to rGO+AgNPs mixtures is dose-dependent and that the addition of controlled concentrations of rGO+Ag nanoparticles on the surfaces of implants may improve the early cellular functions of preosteoblasts, thereby improving the osseointegration of implants while preventing implant-associated infections.

Previous studies have shown the positive effects of other graphene-based materials (e.g., graphene oxide (GO)) on cell viability (L929, MG63, and HFOB cells) [95,96]. GO has been reported to act as an enhancer of mammalian cells by promoting cell adhesion and proliferation [15,96,97,98]. The involved mechanisms have been explored from multiple perspectives—morphological [17,95,99], mechanical [100], and molecular [95,96,101]. However, they have not yet been fully elucidated. Nevertheless, our findings suggest that the presence of the rGO nanosheets supports the adhesion and growth of preosteoblasts, which is known to have a positive impact on early differentiation [100].

The dose-dependent effects observed for the implants incorporating the rGO+Ag mixtures may be related to the toxicity of the Ag^+^ released from the implants, which continued to be released after 28 days (Figure 8) and the cytotoxicity of which largely depends on concentration [102]. Therefore, the beneficial effects of rGO on the growth and early differentiation stage may have been negated by the increasing levels of AgNPs. Therefore, the optimum level of AgNPs in the mixture needs to be determined. Our findings suggest that a mixture of 0.5rGO+0.5AgNPs may be a good starting point for such an optimization study. Apart from the effects of the different rGO and AgNPs loadings, the associated local surface topography with both nano- and submicron features, as revealed by the AFM measurements for the PT-0.5rGO-0.5Ag surface (Figure 7), may have assisted the cell adhesion and spreading of the preosteoblasts, with favorable effects on their osteogenic differentiation [96,97,103].

## 5. Conclusions

rGO, an emerging multifunctional bioactive agent, was combined with AgNPs and incorporated onto the surface of AM porous titanium implants using the PEO method. The incorporation of rGO+Ag mixtures did not significantly affect the PEO voltage transients. The rGO nanosheets were mainly embedded parallel with the implant surface but also orthogonally to the surface, forming ‘nano-knife’ structures around major PEO pores. The dispersion and subsequent incorporation of AgNPs into the PEO layer were improved by the rGO nanosheets. The implants with the addition of rGO showed enhanced antibacterial activity against MRSA compared to the implants incorporating only the AgNPs. This could have been a result of the enhanced Ag incorporation in the presence of rGO, the subsequent release of Ag^+^, the enhanced ROS generation, and the presence of nano-knife structures. The release of Ag^+^ in PBS increased by 31.6% after 28 days, and the initial concentration of ROS rose by 136.9% due to the addition of rGO. Moreover, the same implants (i.e., PT-0.5rGO+0.5Ag) exhibited excellent cytocompatibility for MC3T3-E1 cells and showed the highest stimulatory effect on their osteogenic differentiation. Taken together, the findings of this study revealed a strong antibacterial and osteogenic potential of PEO implants incorporating rGO+Ag nanoparticles that should be further explored. These multifunctional surfaces have high clinical potential for the biofunctionalization of uncemented orthopaedic (3D-printed) titanium implants, where failures caused by antibiotic-resistant bacteria are of tremendous concern.

## Figures and Tables

**Table 1:** Overview of the experimental groups.

Abbreviation	Description
NT	AM Ti-6Al-4V implants
PT	AM Ti-6Al-4V implants oxidized by PEO
PT-0.5rGO	AM Ti-6Al-4V PEO oxidized in electrolytes containing 0.5 g L^−1^ rGO particles
PT-0.5Ag	AM Ti-6Al-4V PEO oxidized in electrolytes containing 0.5 g L^−1^ AgNPs
PT-0.1rGO+0.1Ag	AM Ti-6Al-4V PEO oxidized in electrolytes containing 0.1 g L^−1^ rGO and 0.1 g L^−1^ AgNPs
PT-0.5rGO+0.5Ag	AM Ti-6Al-4V PEO oxidized in electrolytes containing 0.5 g L^−1^ rGO and 0.5 g L^−1^ AgNPs
PT-1.0rGO+1.0Ag	AM Ti-6Al-4V PEO oxidized in electrolytes containing 1.0 g L^−1^ rGO and 1.0 g L^−1^ AgNPs
PT-1.5rGO+1.5Ag	AM Ti-6Al-4V PEO oxidized in electrolytes containing 1.5 g L^−1^ rGO and 1.5 g L^−1^ AgNPs

## Data Availability

Not applicable.
